# Immunogenic cell stress and injury versus immunogenic cell death: implications for improving cancer treatment with immune checkpoint blockade

**DOI:** 10.1080/23723556.2022.2039038

**Published:** 2022-04-03

**Authors:** Ganapathy Sriram, Tiffany R. Emmons, Lauren E. Milling, Darrell J. Irvine, Michael B. Yaffe

**Affiliations:** a David. H. Koch Institute for Integrative Cancer Research, Massachusetts Institute of Technology, Cambridge, MA, USA; b Department of Biological Engineering, Massachusetts Institute of Technology, Cambridge, MA, USA; c Department of Biology, Massachusetts Institute of Technology, Cambridge, MA, USA; d Center for Precision Cancer Medicine, Massachusetts Institute of Technology, Cambridge, MA, USA; e Department of Materials Science and Engineering, Massachusetts Institute of Technology, Cambridge, MA, USA; f The Ragon Institute of Massachusetts General Hospital, Massachusetts Institute of Technology and Harvard University, Cambridge, MA, USA; g Howard Hughes Medical Institute, Chevy Chase, MD, USA; h Divisions of Acute Care Surgery, Trauma, and Surgical Critical Care and Surgical Oncology, Department of Surgery, Beth Israel Deaconess Medical Center, Harvard Medical School, Boston, MA, USA

**Keywords:** Immune checkpoint blockade, DNA damage, immunogenic cell stress, immunogenic cell injury, tumor immunotherapy

## Abstract

Inducing immunogenic tumor cell death to stimulate the response to immune checkpoint blockade has not yet been effectively translated into clinical practice. We recently discovered that stressed/injured but still viable tumor cells are critical for T-cell priming and substantially improve responses to systemic anti-PD1/CTLA4. Therapeutic tumor cell injury, rather than complete killing, in the tumor microenvironment may enhance efficacy of immunotherapy in various cancers.

From a clinical perspective, the ability to combine chemotherapy or radiation treatment with immune checkpoint blockade as a means to enhance cancer treatment responses is highly desirable. The logic behind this approach is simple – reduce the bulk tumor burden and release tumor antigens using conventional standard-of-care cytotoxic treatments because of their high initial anti-tumor response rate, and then use immunotherapy to re-activate preexisting anti-tumor immune cells and prime and expand immune cells against newly released tumor antigens. This would allow the immune system to mop up any residual and/or chemo- or radiation-resistant cancer cells or circulating tumor cells, and confer anti-tumor immunological memory. Translating this rather simple concept of combination chemotherapy with immunotherapy into a uniformly successful clinical paradigm, however, has proven unexpectedly elusive.

To better understand the tumor cell response to cytotoxic injury and the ability of DNA damage signaling in tumors to cross-talk with the immune microenvironment, we explored whether certain regimens of conventional chemotherapeutic drug treatments could stimulate anti-tumor CD8+ T-cell responses by using an optimized tumor cell/dendritic cell/T-cell sequential co-culture system.^1^ We expected to uncover specific types of tumor cell DNA damage and damage-induced signals that induced some form of immunogenic cell death.^2^ Instead, we discovered that strong CD8+ T-cell activation by tumor cells treated with DNA-damaging drugs was highly drug- and dose-dependent and was driven by live stressed/injured tumor cells rather than by dead tumor cells that had undergone any type of cell death - ‘immunogenicʻ, apoptotic, or otherwise. Furthermore, we were able to translate this property of potent adjuvanticity of live stressed/injured tumor cells into a successful pre-clinical treatment protocol in mice using syngeneic models of melanoma and colon carcinoma. When stressed/injured but live tumor cells, generated by etoposide treatment *ex vivo*, were injected directly into primary mouse tumors, in combination with systemic anti-PD1 and anti-CTLA4 antibody treatment, the animals showed marked tumor regression and prolonged survival compared to treatment with anti-PD1/anti-CTLA4 alone. Furthermore, while anti-PD1/CTLA4 alone resulted in no cures, the combination of live stressed/injured tumor cells plus immune checkpoint blockade resulted in a permanent cancer cure in nearly half of the treated animals.

This finding that the live DNA-damaged tumor cells were responsible for the immune-stimulating signal *in vitro* and *in vivo* was unanticipated, and indicates that immunogenic cell ‘deathʻ per se may not be the sine qua non responsible for all potent anti-tumor immune responses. Instead, we refer to this new tumor cell damage phenomenon as “immunogenic cell stress/injury”. Importantly, some tumor cell death was always observed at the dose of the chemotherapeutic drug required to induce immunogenic cell injury in the surviving cells, indicating that a certain threshold of tumor cell damage had to be exceeded in order to induce CD8+ T-cell responses. Directly injecting the DNA damaging agent into solid tumors did not recapitulate the immune-enhancing effect seen with intra-tumoral injections of DNA-damaged live injured tumor cells, suggesting chemotherapy-induced immune cell injury and death within the tumor microenvironment limits the immune response.

According to conventional dogma,^3^ the injured tumor cells should release some type of neo-antigens which would be processed and displayed by DCs as peptides on class I MHC (Signal 1), together with some type of co-stimulatory signal (Signal 2), ideally in the setting of specific proinflammatory cytokines (Signal 3) (Figure 1– illustration ‘aʻ). However, the immune-activating properties of the live injured cells appear to be more complex. First, many of our experiments were performed using OT-I CD8+ T-cells that recognize the SIINFEKL peptide antigen. DCs could theoretically acquire the SIINFEKL antigen through uptake of tumor-derived exosomes,^4^ phagocytosis of dead cell remnants,^5^ or trogocytosis of MHC class I complexes from live injured tumor cells (so-called ‘cross dressingʻ).^6^ However, only live cells, but not dead cells or exosome-containing tumor cell supernatants, were capable of T-cell priming. Furthermore, the tumor cells constitutively expressed the ovalbumin protein with or without damage, but it was only after tumor cell damage that the antigen recognizing T-cells produced IFN-γ and proliferated. In that context at least, SIINFEKL could hardly be considered a classical ‘neoantigenʻ that was only present in the damaged cells, although it is entirely plausible that its level of surface expression may have changed upon damage. Finally, while the majority of T-cell priming was dependent on the presence of DCs, ~30% of the CD8+ T-cell priming was observed when stressed/injured live tumor cells were co-cultured with OT-I CD8+ T-cells even in the complete absence of DCs (unpublished data), indicating that T-cells were capable of directly recognizing the peptide antigen on the surface of the live injured tumor cells, possibly in the presence of some type of stress-induced co-stimulatory signal (Figure 1– illustration ‘bʻ).

**Figure 1.**
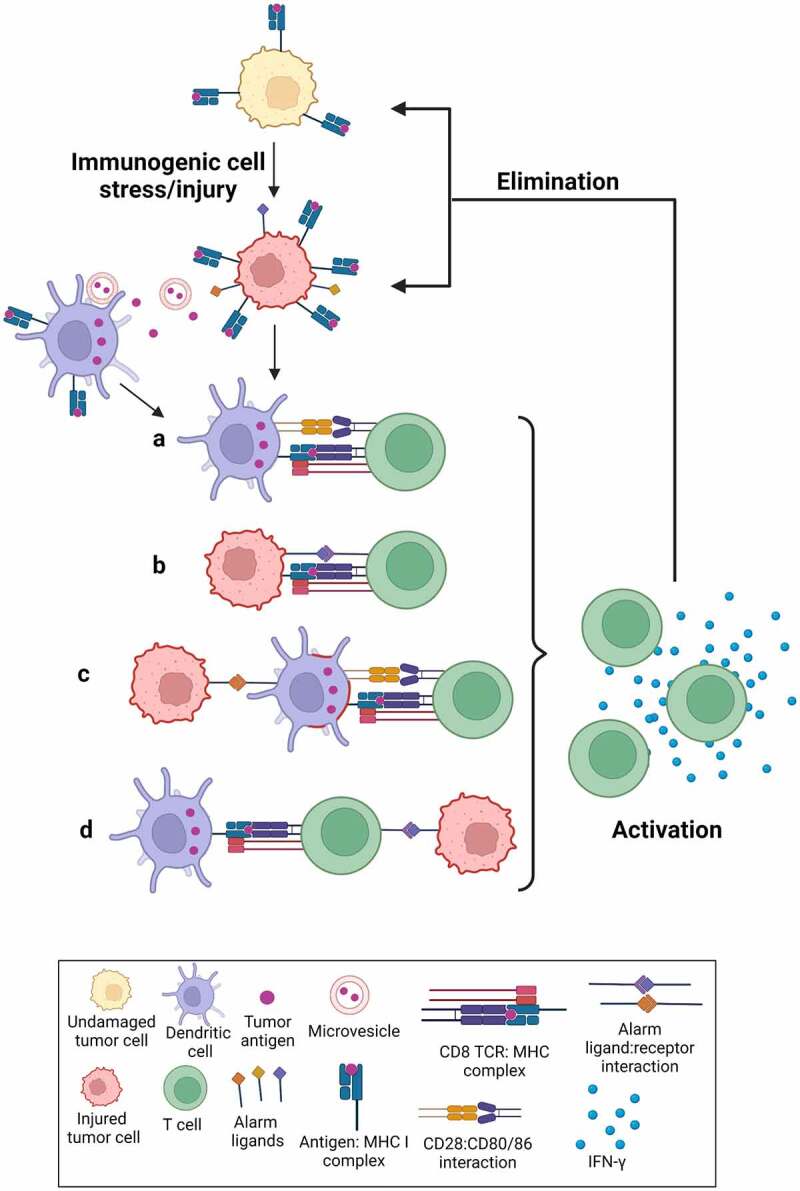
Immunogenic cell stress/injury stimulates an anti-tumor immune response.

Following stress and injury, including chemotherapy-induced DNA damage, antigens from the tumor cells are either released from the cells or displayed on the surface at higher levels, together with stress-activated ligands. In the conventional model (a), DCs cross-present these antigens on MHC-I to prime and activate CD8+ T-cells. A minor but substantial fraction of CD8+ T-cell priming, however, occurs through direct tumor cell:T-cell contact following stress and damage (b). Finally, our observation that only live stressed/injured cells were capable of driving DC-mediated T-cell priming indicates that antigen delivery to, and/or stimulation of DCs involves direct tumor cell:DC contact (c), and even allows the possibility of a 3-way interaction between the tumor cells, DCs, and T-cells (d). Figure created with BioRender.com.

Our finding that DNA damaging agents can induce a state of immunogenic cell stress/injury within tumors is in good agreement with, and extends later versions of the Danger theory of immune function proposed by Matzinger.^7^ In the context of anti-tumor immunity, the Danger model posits that stimulation of an immune response requires an element of cell stress or damage that causes the expression of alarm signals by tumor cells. Since culture supernatants or cell lysates from damaged/stressed cells did not induce a T-cell response, the alarm signal sensed by DCs likely resides on the surface of live injured tumor cells, and is created through activation of stress signaling pathways. This is supported by our data that active signaling through several common stress signaling pathways: NF-KB, ATR, DNA-PK, p38MAPK and RIPK1, contributes to this response.

We propose a model wherein “immunogenic cell stress/injury” to tumor cells activates signaling pathways that ultimately increase the expression of damage/stress-associated surface molecules^8^ which either enhance antigen cross-presentation by DCs, or directly form ligands for T-cell co-stimulation (Figure 1). We specifically note that the tumor antigen (Signal 1), damage/stress-associated co-stimulatory signals (Signal 2) and soluble mediators (Signal 3) necessary for T-cell priming/activation could potentially come from different cell types (Figure 1– illustrations ‘cʻ and ‘dʻ).

Clinical translation of this immunogenic cell injury approach to therapy can be conceptualized in at least the following three ways: 1) Induction of immunogenic cell stress/injury to patient-derived tumor cells *ex vivo* followed by injecting these live injured tumor cells directly into the tumor in combination with systemically administered immune checkpoint blockade therapy; 2) Induction of immunogenic tumor cell stress/injury by direct application of specific stressors (for e.g. cryotherapy or radiation) to tumors *in situ* in the neo-adjuvant setting, combined with systemic immune checkpoint blockade therapy; or 3) Using antibody-drug conjugates to specifically deliver immunogenic stress/injury-inducing drugs to tumor cells while avoiding injury to immune cells within the tumor microenvironment. Each of these approaches would require identifying a treatment for tumor cells that induces immunogenic stress/injury which might vary in identity, dose and/or timing depending on the tumor type. Additional knowledge of surface molecules that are displayed on tumor cells following immunogenic cell injury could serve as a biomarker of response, and would have a major impact on translating these findings into the clinic.
